# Impact of Semaglutide, Liraglutide and Tirzepatide on Cardiometabolic Outcomes: A Comparative Narrative Review

**DOI:** 10.1002/edm2.70338

**Published:** 2026-09-16

**Authors:** Hiba Bawadi, Haya Abuhijleh, Mamon Nofal, Zain Z. Zakaria, Maha Al‐Asmakh

**Affiliations:** ^1^ Department of Human Nutrition College of Health Sciences, QU‐HEALTH, Qatar University Doha Qatar; ^2^ Clinical Affairs Department QU‐HEALTH, Qatar University Doha Qatar; ^3^ Department of Biomedical Sciences College of Health Sciences, QU‐HEALTH, Qatar University Doha Qatar; ^4^ Nottingham University Hospitals NHS Trust Nottingham UK; ^5^ Biomedical Research Center QU‐HEALTH, Qatar University Doha Qatar

**Keywords:** cardiovascular outcomes, lipid modulation, liraglutide, obesity, renal protection, semaglutide, tirzepatide, type 2 diabetes

## Abstract

**Background:**

Cardiometabolic disorders such as type 2 diabetes mellitus (T2DM), obesity and cardiovascular disease (CVD) remain major global health challenges. Incretin‐based therapies, including the GLP‐1 receptor agonists semaglutide and liraglutide, and the dual GIP/GLP‐1 receptor agonist tirzepatide, are increasingly used to address hyperglycaemia and obesity, and have been evaluated for both cardiometabolic risk markers and selected cardiovascular and renal outcomes.

**Objective:**

This review summarizes and contrasts the pharmacology of semaglutide, liraglutide and tirzepatide, distinguishing between surrogate cardiometabolic risk markers (e.g., glycaemic control, body weight and lipid parameters) and confirmed clinical outcomes, including major adverse cardiovascular events (MACE) and renal endpoints.

**Methods:**

Recent data were synthesized from pivotal randomized clinical trial programs, SUSTAIN and PIONEER (semaglutide), LEADER (liraglutide) and SURPASS and SURMOUNT (tirzepatide), as well as meta‐analyses, post hoc analyses and observational studies addressing mechanistic and long‐term cardiometabolic outcomes.

**Results:**

All three agents improved HbA1c, weight and cardiometabolic outcomes, with tirzepatide generally showing greater reductions across clinical trials. Semaglutide and liraglutide consistently improved BMI, lipid profile and cardiovascular risk, while tirzepatide shows promise pending CVOT data. Renal outcomes were favourable across the class, though results varied by baseline characteristics and trial design.

**Conclusion:**

Semaglutide, liraglutide and tirzepatide are key therapies for type 2 diabetes and cardiometabolic disease. Tirzepatide's dual activity may enhance metabolic and lipid outcomes, while semaglutide and liraglutide have strong evidence for cardiovascular and renal safety. However, comparisons are based on indirect evidence and should be interpreted cautiously.

## Graphical abstract

The comparative pharmacology and cardiometabolic outcomes of semaglutide, liraglutide and tirzepatide, integrating evidence from major clinical trial programs and meta‐analyses.

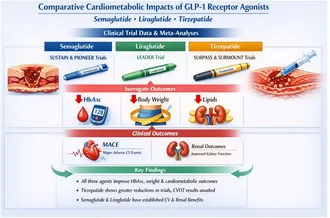

## 1 Introduction

Cardiometabolic disorders, including type 2 diabetes mellitus (T2DM), obesity and cardiovascular disease (CVD), pose a major global health challenge, accounting for a significant share of morbidity and mortality [1]. Cardiovascular diseases alone contribute to approximately 31% of global deaths, totaling 17.9 million annually, with projections exceeding 23.6 million by 2030 [1, 2]. T2DM and obesity are closely linked to cardiovascular health, with obesity—particularly visceral adiposity—strongly associated with elevated cardiometabolic risk [3].

The convergence of obesity and T2DM substantially amplifies cardiovascular risk and renal disease progression, creating a clinical need for therapies that improve glycaemic control and body weight reduction while also influencing cardiovascular and kidney outcomes.

Incretin‐based therapies have emerged as important pharmacologic agents in this context. Glucagon‐like‐peptide‐1 receptor agonists (GLP‐1 RAs), including semaglutide and liraglutide, and the dual glucose‐dependent insulinotropic polypeptide/glucagon‐like peptide‐1 receptor agonist (GIP/GLP‐1 RA) tirzepatide have demonstrated benefits across several cardiometabolic domains. These include improvements in glycaemic control, body weight and cardiometabolic risk markers such as lipid profiles and renal function [4, 5]. In addition, evidence from cardiovascular outcome trials (CVOTs) indicates a lower risk of major adverse cardiovascular events for certain agents within this class [6, 7, 8, 9].

Despite these advances, direct head‐to‐head comparisons between these agents across similar populations and dosing regimens remain limited. As a result, current evidence is largely derived from separate clinical trial programs with heterogeneous designs, populations and endpoints. This creates challenges in interpreting comparative efficacy and clinical positioning across agents.

Therefore, this review aims to provide a comparative narrative synthesis of semaglutide, liraglutide and tirzepatide, focusing on glycaemic control, weight reduction, lipid profiles, cardiovascular outcomes and renal markers. Particular emphasis is placed on distinguishing between surrogate cardiometabolic risk markers and confirmed clinical outcomes, while acknowledging the limitations of indirect comparisons across studies. This approach is intended to support clinicians in aligning therapeutic choices with individual patient characteristics and treatment goals.

## 2 Methods

This manuscript was conducted as a structured comparative narrative review. The review approach was guided by established principles for high‐quality narrative synthesis, aligned with the Scale for the Assessment of Narrative Review Articles (SANRA), to ensure a clear focus, transparent study selection and balanced interpretation of evidence [10].

A comprehensive literature search was conducted across PubMed, Scopus and Google Scholar through October 2025 using terms related to incretin‐based therapies, diabetes, obesity, cardiovascular and renal outcomes and lipid profiles. Reference lists of pivotal trials and reviews were also screened to capture key publications.

Eligible studies included randomized controlled trials, supporting meta‐analyses, pooled analyses and selected post hoc or observational studies published in English that assessed metabolic or cardiorenal outcomes. Evidence was prioritized from pivotal randomized phase 3 programs, SUSTAIN and PIONEER (semaglutide), LEADER (liraglutide), and SURPASS, SURMOUNT (tirzepatide), meta‐analyses, and pooled analyses, while post hoc or observational studies were used as supportive/contextual evidence.

Data extraction focused on key study characteristics, including population, intervention, comparator, dose, duration and reported cardiometabolic outcomes. Findings were synthesized qualitatively and organized by outcome domains (glycaemic control, weight, cardiovascular, renal and lipid outcomes) to enable structured comparison across agents.

Given the absence of direct comparative trials and the heterogeneity in study design, populations and outcome definitions, no formal quantitative comparison or meta‐analysis was performed. Differences across studies were considered when interpreting findings.

## 3 Pharmacological Profiles of Semaglutide, Tirzepatide and Liraglutide: Mechanistic and Clinical Distinctions

**FIGURE 1:**
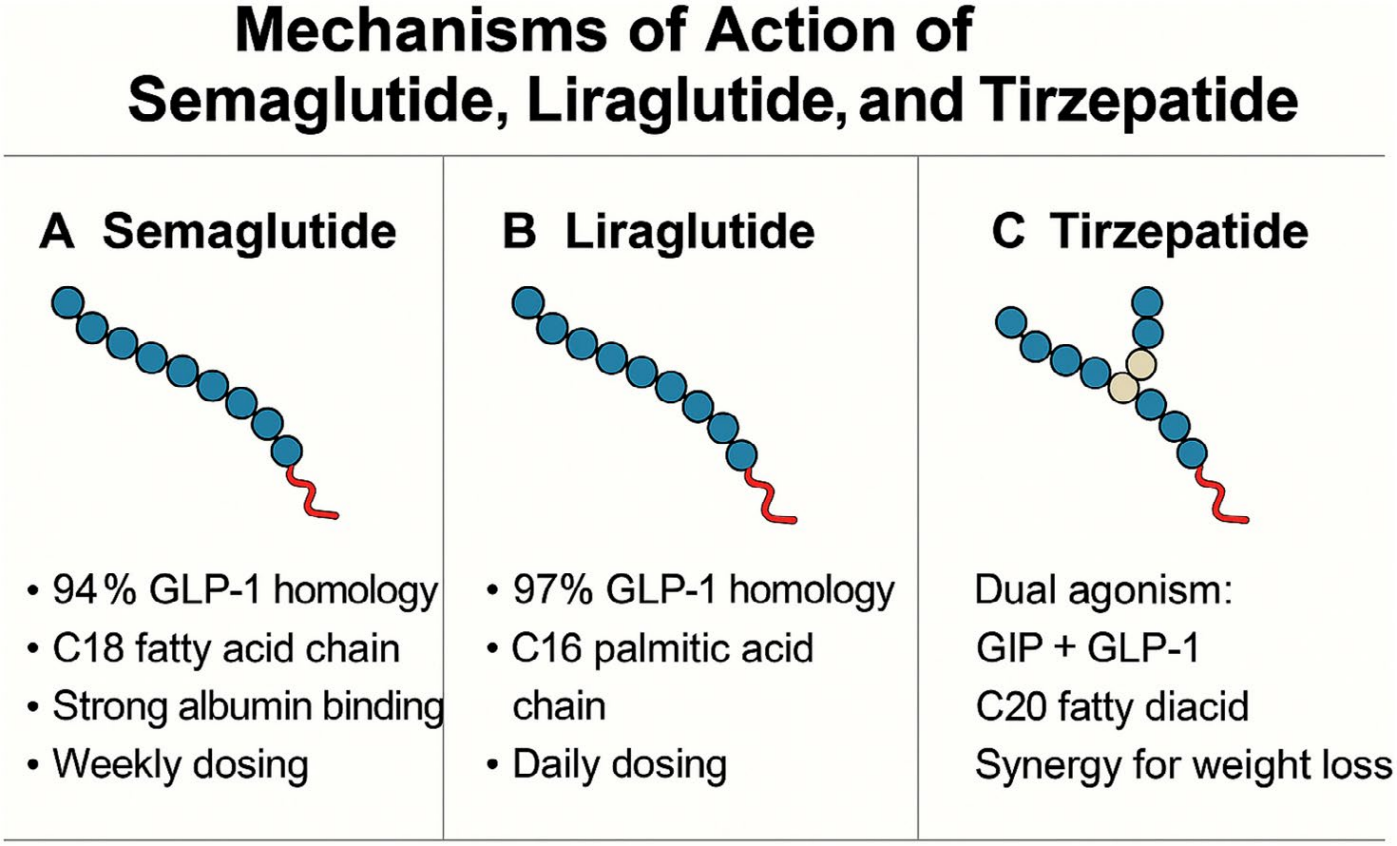
Mechanisms of Action of semaglutide, liraglutide and tirzepatide. Panel (A) depicts semaglutide, a GLP‐1 receptor agonist containing a C18 fatty diacid side chain that enhances albumin binding and permits once‐weekly administration. Panel (B) illustrates liraglutide, a GLP‐1 receptor agonist with a C16 palmitic acid side chain supporting once‐daily dosing. Panel (C) presents tirzepatide, a dual GIP/GLP‐1 receptor agonist incorporating a C20 fatty diacid moiety that extends systemic exposure. Collectively, the panels highlight the structural modifications underlying the distinct pharmacokinetic behaviour and metabolic effects of these agents. Detailed data sources and methodological notes for this figure are provided in File S1.

Distinct structural modifications underpin the pharmacological behaviour and clinical applications of semaglutide, liraglutide and tirzepatide. Semaglutide, a human GLP‐1 analog with 94% sequence identity to endogenous GLP‐1, incorporates an Aib substitution at position 8 to confer resistance to DPP‐4 degradation and a C18 fatty diacid side chain at position 26 to facilitate albumin binding and extend half‐life, enabling once‐weekly dosing [11, 12]. Liraglutide has 97% sequence homology to native GLP‐1 and employs a C16 palmitic acid side chain to promote albumin binding, though its shorter chain and lower hydrophobicity result in a half‐life that supports only once‐daily administration [11]. Tirzepatide, a 39‐amino acid synthetic ‘twincretin’, acts as a dual GIP and GLP‐1 receptor agonist; it features a C20 fatty diacid moiety that enhances albumin affinity and prolongs systemic exposure, delivering synergistic insulinotropic and anorexigenic effects [12] (Table 1), A visual summary of these structural and receptor differences is provided in Figure 1.

**TABLE 1:** Comparative pharmacological properties, clinical characteristics and therapeutic profiles of semaglutide, liraglutide and tirzepatide.

Category	Semaglutide	Liraglutide	Tirzepatide
Drug class	GLP‐1 receptor agonist	GLP‐1 receptor agonist	Dual GLP‐1/GIP receptor agonist (‘twincretin’)
Sequence homology	94% to human GLP‐1	97% to human GLP‐1	Synthetic sequence
Key structural modifications	Aib at position 8; C18 fatty diacid at position 26	C16 palmitic acid chain	C20 fatty diacid moiety
Function of modifications	DPP‐4 resistance, albumin binding, prolonged half‐life	Promotes albumin binding	Prolonged albumin binding and systemic exposure
Route of administration	Subcutaneous (weekly); Oral (daily, SNAC‐mediated)	Subcutaneous only	Subcutaneous only
Formulation	Prefilled pen; oral tablet	Prefilled multi‐dose pen	Prefilled autoinjector
Dosing frequency	Weekly (SC); Daily (oral)	Daily (SC)	Weekly (SC)
Half‐life	~7 days	~13 h	~5 days
Bioavailability (SC)	~89%	~55%	Not published
Bioavailability (oral)	~1% (via SNAC)	N/A	N/A
Albumin binding	Strong (C18 side chain)	Moderate (C16 chain, reversible)	Strong (C20 side chain)
Renal impairment use	Stable in mild/moderate; variability in ESRD—caution advised	Stable in mild/moderate; variability in ESRD—caution advised	Stable across all stages, including dialysis— advantageous
Hepatic impairment use	Stable across all stages	Use with caution in severe hepatic dysfunction (limited data)	Stable across all stages
Delivery & storage	SC pen and oral tablet; good room‐temp stability; oral form requires fasting and water‐only intake	SC daily pen; stable	SC autoinjector; stable
Adherence factors	Flexible options; ~40% persistence at 1 year	Daily injections may reduce adherence	High adherence expected due to weekly dosing; real‐world data pending
Tolerability	Dose‐dependent GI effects (nausea, vomiting, diarrhoea); subside in 4–8 weeks	Generally milder GI symptoms with gradual onset	Higher frequency and severity of GI effects (esp. constipation); GIP‐related
Unique advantages	Only oral GLP‐1 RA; highest persistence; weekly or daily options	Approved for adolescent use (T2DM & obesity); better tolerated in some patients	Strongest glycaemic/weight effect; stable in ESRD; ideal for obesity/GLP‐1 failure

Abbreviations: ESRD, end‐stage renal disease; SC, subcutaneous.

The molecular characteristics of the three GLP‐1 RA agents dictate the route and frequency of administration (Figure 1). Semaglutide stands out as the only GLP‐1 receptor agonist available in both subcutaneous and oral forms—the latter utilizing SNAC (sodium N‐[8‐(2‐hydroxybenzoyl) amino] caprylate) to facilitate transcellular gastric absorption and allow once‐daily tablet dosing [13, 14]. By comparison, liraglutide requires once‐daily subcutaneous administration via a multi‐dose prefilled pen, aligning with its shorter pharmacokinetic duration [11]. Tirzepatide, formulated exclusively as a once‐weekly subcutaneous autoinjector, leverages its extended half‐life to maximize convenience [12].

Pharmacokinetically, these agents diverge in half‐life, bioavailability and systemic exposure. Semaglutide's 7‐day half‐life supports both weekly subcutaneous and daily oral dosing, though the latter achieves only 1% bioavailability, aided by SNAC‐mediated absorption. Subcutaneous semaglutide achieves ~89% bioavailability through stable albumin binding [14, 15]. Liraglutide's half‐life of 13 h, combined with 55% subcutaneous bioavailability, reflects its self‐association into heptamers and reversible albumin binding, necessitating daily use [11]. Tirzepatide similarly benefits from albumin conjugation via its C20 fatty acid, yielding a 5‐day half‐life despite unpublished absolute bioavailability data.

In terms of use in renal and hepatic impairment, these agents exhibit favourable pharmacokinetics. All three are primarily degraded by proteolysis, with minimal renal or hepatic elimination, allowing stable exposure in mild to moderate dysfunction [14, 16]. However, in ESRD or severe renal impairment, exposure variability may rise for semaglutide and liraglutide, warranting clinical caution [14]. Tirzepatide maintains consistent pharmacokinetics across all stages of renal impairment, including dialysis, offering a practical advantage for this high‐risk population [12, 17]. Similarly, while liraglutide should be used cautiously in severe hepatic dysfunction due to limited evidence, both semaglutide and tirzepatide maintain stable profiles across hepatic function grades, as confirmed by pharmacokinetic trials and regulatory reviews [12, 14].

Delivery systems are optimized across the class to reduce user burden and improve accuracy. All three drugs are available as prefilled pens with reliable stability profiles and defined room‐temperature tolerability [11, 12, 14]. Semaglutide's unique oral formulation expands its accessibility, though it requires fasting and water‐only intake due to low (~1%) oral bioavailability [14, 18]. While liraglutide's daily injection may deter some patients, tirzepatide's once‐weekly dosing supports improved adherence in many clinical settings [12, 17]. Real‐world data show that semaglutide achieves approximately 40% treatment persistence at 1 year, higher than liraglutide, reflecting its efficacy, flexible formulation and reduced injection burden [19]. Tirzepatide is pending further real‐world outcomes.

Tolerability and adverse effects further inform agent selection. Gastrointestinal symptoms: nausea, vomiting, diarrhoea are common across all three, though severity and onset differ [12, 13, 14]. Semaglutide's effects are dose‐related and usually subside within 4–8 weeks with proper titration [14]. Tirzepatide exhibits more frequent and intense GI effects, including constipation, especially at higher doses, likely attributable to its GIP receptor activity [12]. Despite this, it provides superior glycemic and weight reduction, making it ideal for patients with obesity, insulin resistance, or prior GLP‐1 monotherapy failure [12, 17]. Liraglutide, although better tolerated in terms of gradual GI effects, is limited by daily dosing and more modest metabolic outcomes. Nonetheless, it remains appropriate for patients accustomed to daily injections or requiring flexible paediatric indications, as it is the only agent approved in adolescents with both diabetes and obesity [11, 20].

## 4 Comparative Impact on Cardiometabolic Risk Factors

### 4.1 Type 2 Diabetes

Glycemic control remains a cornerstone in the management of T2DM, with HbA1c serving as a clinically validated biomarker for long‐term glucose regulation [21]. HbA1c reflects the average blood glucose concentration over approximately 2–4 months, capturing chronic glycemic exposure and its association with long‐term complications [21]. HbA1c is consistently employed as a primary efficacy endpoint in pivotal diabetes trials, including those evaluating GLP‐1 receptor agonists and dual incretin therapies [22, 23, 24]. Its widespread adoption in regulatory guidance and clinical trial design underscores its central role in assessing therapeutic efficacy and guiding treatment decisions in T2DM management [21]. This section serves as a comparative analysis on the effect of semaglutide, liraglutide and tirzepatide on HbA1c as a central glycemic endpoint across their respective clinical development programs.

#### 4.1.1 Semaglutide

Semaglutide, a GLP‐1 receptor agonist, has demonstrated efficacy in reducing HbA1c across both subcutaneous (injectable) and oral formulations [6, 25, 26]. Subcutaneous (SC) semaglutide has demonstrated meaningful reductions in HbA1c levels in multiple randomized clinical trials within the SUSTAIN program [27, 28, 29, 30, 31]. In the SUSTAIN 1 trial, once weekly SC semaglutide at doses of 0.5 mg and 1.0 mg reduced HbA1c by 1.45% and 1.55%, respectively, over 30 weeks compared with a 0.10% reduction in the placebo group [28]. In SUSTAIN 2, semaglutide 0.5 mg and 1.0 mg led to reductions of 1.30% and 1.60% over 56 weeks, outperforming sitagliptin which achieved a 0.50% reduction [29]. The SUSTAIN 3 trial showed a 1.50% HbA1c reduction with semaglutide 1.0 mg administered subcutaneously over 56 weeks, which was significantly greater than the 0.90% reduction achieved with extended release exenatide [30]. In SUSTAIN 4, semaglutide 0.5 mg and 1.0 mg SC achieved reductions of 1.21% and 1.64%, respectively, compared to 0.83% with insulin glargine over 30 weeks [31]. Finally, SUSTAIN 5 reported HbA1c reductions of 1.40% and 1.80% with semaglutide 0.5 mg and 1.0 mg added to basal insulin, versus 0.10% with placebo over 30 weeks [27].

These trials consistently demonstrate that once‐weekly SC semaglutide at doses of 0.5 mg and 1.0 mg achieves clinically meaningful reduction in HbA1c ranging from 1.3% to 1.8%, with superior glycemic efficacy compared with placebo, other GLP‐1 receptor agonists and basal insulin [27, 28, 29, 30, 31]. Efficacy was maintained across diverse patient populations and baseline characteristics, reinforcing semaglutide's role as a potent agent in T2DM management.

Oral semaglutide, evaluated in the randomized PIONEER trials, has consistently demonstrated reductions in HbA1c across diverse patient populations and dosing regimens, often outperforming established comparators including liraglutide. In the PIONEER 1 trial, oral semaglutide monotherapy led to HbA1c reductions of up to 1.4% over 26 weeks compared to placebo [32]. In the PIONEER 3 trial, participants treated with 7 mg and 14 mg of oral semaglutide exhibited HbA1c reductions of 1.3% after 26 weeks, outperforming sitagliptin (a DPP‐4 inhibitor), which showed more modest reductions [33]. In the PIONEER 4 trial, oral semaglutide 14 mg demonstrated a greater glycemic efficacy at week 26 compared to subcutaneous liraglutide 1.8 mg, with an estimated treatment difference (ETD) in HbA1c reduction of −0.2% [34]. Observational data from retrospective analysis have also reported HbA1c reductions of up to 2.0% in patients with poorly controlled type 2 diabetes mellitus treated with oral semaglutide, supporting its effectiveness in glycemic control in routine clinical practice [35].

#### 4.1.2 Liraglutide

The efficacy of liraglutide in managing type 2 diabetes mellitus, particularly its impact on glycated haemoglobin levels, has been extensively evaluated across randomized clinical trials, notably in the LEAD (Liraglutide Effect and Action in Diabetes) trials. These trials confirm that liraglutide reduces HbA1c levels when administered at doses of 1.2 mg and 1.8 mg SC. Reported reductions in HbA1c span from 0.7% to 1.5%, showcasing liraglutide's capability to achieve glycemic control effectively [36, 37, 38, 39, 40].

In the LEAD‐2 study, once daily SC liraglutide administered at doses of 1.2 mg and 1.8 mg administered for 26 weeks in combination with metformin achieved mean HbA1c reductions of 1.0%, comparable to glimepiride, whereas the 0.6 mg dose reduced HbA1c by 0.7% and placebo was associated with a 0.1% increase [38]. The LEAD‐3 trial, which evaluated liraglutide as monotherapy over 52 weeks, reported HbA1c reductions of 0.84% with 1.2 mg and 1.14% with 1.8 mg, compared to 0.51% with glimepiride, confirming its superior glycemic control [37].

In LEAD‐4, liraglutide combined with metformin and rosiglitazone over 26 weeks achieved HbA1c reductions of 1.5% for both 1.2 mg and 1.8 mg SC doses, which were significantly greater than the 0.5% reduction observed with placebo [40]. The LEAD‐5 trial compared liraglutide to insulin glargine and placebo over 26 weeks, showing a 1.33% HbA1c reduction with liraglutide 1.8 mg, versus 1.09% with insulin glargine and 0.24% with placebo [39]. Finally, LEAD‐6 demonstrated liraglutide's superiority over exenatide, with a 1.12% HbA1c reduction at 26 weeks compared to 0.79% with exenatide, alongside improved tolerability and fewer gastrointestinal side effects [36].

Evidence from systematic reviews and real‐world observational studies similarly indicates that liraglutide reduces HbA1c levels by approximately 0.9%–2.2% in clinical practice [41, 42]. Moreover, one study demonstrated that the reduction of HbA1c aligns with the LEAD trials, illustrating a real‐world effectiveness that mirrors outcomes observed in controlled clinical trial environments [43]. Beyond monotherapy, combining liraglutide with basal insulin (iDegLira) has shown superior real‐world efficacy over premixed insulin regimens, significantly increasing continuous glucose monitoring (CGM) time in range (TIR) and lowering HbA1c alongside a near‐halving of the total daily insulin dose [44].

A head‐to‐head comparison within the LEAD framework has demonstrated that liraglutide reduced HbA1c to a greater extent than several comparators, including other GLP‐1 receptor agonists and sitagliptin, positioning it as an effective option in type 2 diabetes management [45]. This observation is also reflected in a retrospective cohort study, indicating that patients initiating liraglutide therapy have a higher likelihood of achieving HbA1c targets compared to those receiving alternative treatments [46].

Furthermore, in a retrospective observational study, the reduction in HbA1c through liraglutide is reportedly independent of weight loss; however, there is a noted association wherein greater reductions in HbA1c often correlate with higher baseline HbA1c values [47]. This is crucial in tailoring diabetes treatment to individual patient profiles.

##### 4.1.2.1 Tirzepatide

Tirzepatide, a novel dual GIP and GLP‐1 receptor agonist, has gained significant attention in clinical research for its efficacy in managing T2DM. The SURPASS clinical trials, comprising SURPASS‐1 through SURPASS‐5, systematically evaluate tirzepatide's effects at varying SC dosages (5 mg, 10 mg and 15 mg) over treatment periods ranging from 40 to 52 weeks, demonstrating significant drops in HbA1c levels across all studies [48, 49]. The results from these randomized clinical trials indicate that tirzepatide consistently achieves reductions in HbA1c when compared to established treatments such as semaglutide and insulin [17, 50].

For instance, in the randomized controlled trial SURPASS‐2, tirzepatide at doses of 5 mg, 10 mg and 15 mg led to mean HbA1c reductions of approximately 2.01%, 2.24% and 2.30%, respectively, from baseline. In comparison, semaglutide 1 mg resulted in a reduction of about 1.86% [17]. Across all doses, in the SURPASS‐2 trial, tirzepatide demonstrated greater HbA1c reductions compared with semaglutide 1 mg under the specific conditions of this study.

Pooled analyses of SURPASS randomized trials show that more than 90% of tirzepatide recipients achieved an HbA1c target of < 7.0% [49]. Moreover, the most substantial reductions were observed in patients with higher baseline HbA1c levels, reinforcing tirzepatide's effectiveness across diverse patient populations [51, 52].

Tirzepatide demonstrated sustained glycaemic control, with additional effects on body weight and other cardiometabolic risk markers [53, 54]. Across 40‐week trials, the median proportion of treatment duration spent with HbA1c < 7.0% was 80% with tirzepatide, compared to 70% with semaglutide and 0% with placebo. In 52‐week studies, this ranged from 77% to 85% with tirzepatide, compared to 62% with insulin degludec and 23% with insulin glargine (*p* < 0.001) [49]. Notably, time in the target HbA1c range was consistent across tirzepatide doses. Tirzepatide‐treated participants also spent a longer duration achieving both HbA1c ≤ 6.5% and ≥ 5% body weight reduction compared to semaglutide (median: 35%–60% vs. 7%; *p* < 0.001), underscoring its dual‐action therapeutic potential and a comprehensive approach to diabetes management [49, 54]. While these findings highlight combined glycaemic and weight effects, they should be interpreted within the context of glycaemic outcome‐focused analyses.

Additionally, post hoc analyses derived from the SURPASS trial cohorts demonstrated that a greater proportion of tirzepatide‐treated individuals with T2D achieved composite endpoints combining glycaemic control, varying degrees of weight loss and absence of hypoglycaemia compared to comparator groups [55]. Furthermore, tirzepatide was associated with significant reductions in both HbA1c and body weight irrespective of GADA status, suggesting potential benefit in individuals with latent autoimmune diabetes in adults (LADA) [56]. These findings are derived from post hoc analyses and should be interpreted as supportive evidence.

In summary, semaglutide, liraglutide and tirzepatide have each demonstrated reductions in HbA1c among individuals with type 2 diabetes mellitus, with varying degrees of efficacy and trial durations. Subcutaneous and oral semaglutide achieved reductions between 1.3% and 2.0%, while liraglutide showed improvements ranging from 0.7% to 1.5%. Tirzepatide, as a dual GIP and GLP‐1 receptor agonist, generally showed greater HbA1c reductions, reaching up to 2.30% in some trials.

These findings are derived from independent clinical trial programs with differing study designs, populations and comparators, and should therefore be interpreted as indirect and non‐comparative rather than definitive evidence of superiority between agents.

From a clinical perspective, these differences may be relevant when selecting therapy for patients requiring more intensive glycaemic control or rapid achievement of target HbA1c levels, particularly in individuals with higher baseline HbA1c or those inadequately controlled on existing therapies, where agents associated with greater HbA1c reductions may provide incremental benefit.

**FIGURE 2:**
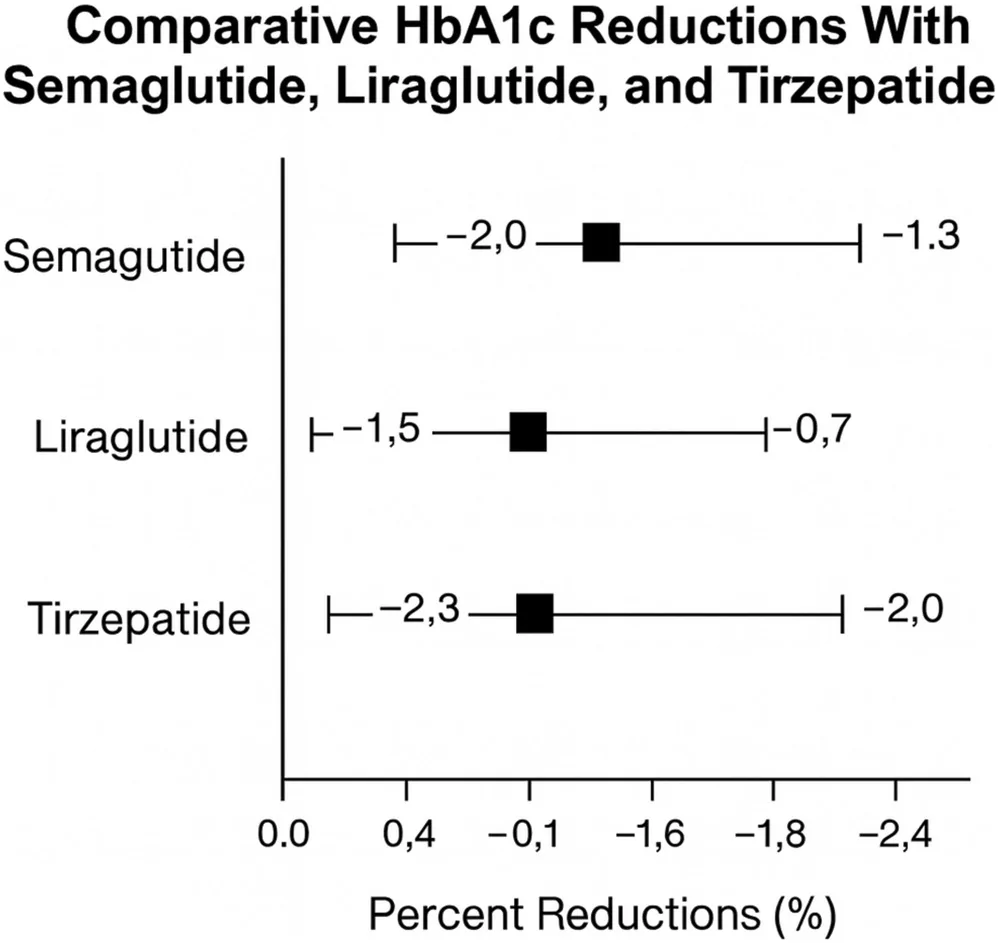
Comparative HbA1c reductions with semaglutide, liraglutide and tirzepatide. Forest plot illustrates the range of HbA1c reductions reported in pivotal clinical trials of semaglutide, liraglutide and tirzepatide. Semaglutide achieved reductions of approximately −1.3% to −2.0%, liraglutide produced reductions of −0.7% to −1.5%, and tirzepatide demonstrated the largest reductions, ranging from −2.0% to −2.3%. Estimates are derived from the SUSTAIN and PIONEER (semaglutide), LEAD (liraglutide) and SURPASS (tirzepatide) clinical development programs. Detailed data sources and methodological notes for this figure are provided in File S1, Figure S1.

Table 2 summarizes the impact of semaglutide, liraglutide and tirzepatide on HbA1c reduction as reported in the SUSTAIN, PIONEER, LEAD and SURPASS clinical trials, highlighting their comparative efficacy in glycemic control among individuals with type 2 diabetes mellitus (Table 2), A visual comparison of HbA1c reductions across the three agents is presented in Figure 2.

**TABLE 2:** Comparative summary of HbA1c reduction with semaglutide, liraglutide and tirzepatide across major phase 3 clinical trial programs (SUSTAIN, PIONEER, LEAD and SURPASS).

Agent	Trial	Dose (mg)	HbA1c reduction (%)	Duration (weeks)
Semaglutide				
Subcutaneous	SUSTAIN	0.5–1.0	1.3–1.8	30–56
Oral	PIONEER	7–14	1.3–2.0	26
Liraglutide	LEAD	1.2–1.8	0.7–1.5	26–52
Tirzepatide	SURPASS	5–15	2.01–2.30	40–52

### 4.2 Weight Reduction

Weight reduction is a key therapeutic goal in the management of type 2 diabetes and obesity. A weight loss of 5%–10% is generally considered clinically meaningful in improving metabolic outcomes and reducing obesity‐related risks [57]. Weight, body mass index (BMI), and waist circumference are key indicators for evaluating weight loss and related health outcomes [58, 59]. They are especially relevant in obesity management, including bariatric surgery and lifestyle interventions. Studies show that greater weight reduction improves metabolic health and lowers obesity‐related risks [58, 59]. Waist circumference, in particular, is a stronger predictor than BMI in certain populations, highlighting its clinical value in assessing abdominal obesity [55, 60]. These measures are routinely used in randomized clinical trials and observational studies examining the effects of GLP‐1 receptor agonists and dual incretin therapies on weight loss.

#### 4.2.1 Semaglutide

Clinical trials have reported that semaglutide is associated with reductions in body weight and BMI. In the randomized STEP 4 trial, participants receiving 2.4 mg SC semaglutide experienced an average weight loss of approximately 10.6% from baseline [61]. The randomized STEP trials, including STEP 1 and STEP 4, reported mean weight reductions of up to 14.9% at 68 weeks compared to 2.4% in placebo. Similarly, the SELECT trial demonstrated that once‐weekly 2.4 mg SC semaglutide led to a mean weight reduction of 10.2% over 208 weeks, along with a decrease in waist circumference (−7.7 cm), suggesting potential benefits in reducing visceral adiposity (*p* < 0.0001) [56]. These effects were observed across various subgroups, including different sexes, BMI categories and geographic regions. Although treatment discontinuation rates were higher among participants with lower BMI, the incidence of serious adverse events was lower in the semaglutide group [56].

The weight‐lowering effects of semaglutide were observed across both subcutaneous (0.5–1.0 mg weekly) and oral formulations in the SUSTAIN and PIONEER trials, respectively, as well as in the STEP program, which evaluated a 2.4 mg weekly dose in individuals with obesity. Across these studies, semaglutide demonstrated greater reductions in weight and BMI compared to placebo and other antidiabetic agents [62].

Notably, participants consistently maintained BMI reductions, with many transitioning from higher to lower BMI categories over time [62]. In a STEP trial, over 40% of participants transitioned to a BMI category below the obesity threshold during treatment, suggesting potential utility in long‐term weight management [63]. Moreover, observational real‐world studies support these findings, with Wajid et al. [64] reporting a mean BMI reduction of approximately 1.5 kg/m^2^ in patients with type 2 diabetes treated with semaglutide, while Qin et al. [65] observed a greater mean BMI decline of 4.5 kg/m^2^ in a meta‐analysis of randomized trials using the 2.4 mg dose, highlighting high efficacy across clinical settings [64, 65].

#### 4.2.2 Liraglutide

Liraglutide has also been evaluated for its effects on weight, BMI and waist circumference in individuals with type 2 diabetes, particularly in the randomized LEAD clinical trial program [66, 67]. Its mechanisms of action include delayed gastric emptying, appetite suppression and enhanced satiety, which may contribute to reduced caloric intake and subsequent weight loss [68].

In LEAD‐2, liraglutide at 0.6 mg, 1.2 mg and 1.8 mg SC was associated with a mean weight reduction of approximately 2.4 kg (≈5.3 lbs) compared to a small increase of approximately 1.0 kg in those treated with glimepiride [66]. A meta‐analysis of clinical trials reported that liraglutide at a dose of 3.0 mg/day can facilitate a mean body weight reduction of approximately 4.91 kg, a BMI decrease of 1.86 kg/m^2^, and a waist circumference reduction of 3.55 cm, irrespective of glycemic status [68]. These effects were consistent across individuals with varying baseline BMIs, with greater absolute reductions observed among those with higher initial obesity class [68]. Additionally, expanding therapeutic accessibility, a Phase III post hoc evaluation of a liraglutide biosimilar (up to 1.8 mg) in Indian patients with type 2 diabetes and obesity demonstrated significant weight reductions ranging from 5.5 to 7.1 kg over 24 weeks, comparable to those achieved with the reference liraglutide, thereby confirming its clinical non‐inferiority [69].

Also, liraglutide administration at 0.3, 0.6 and 0.9 mg resulted in a progressive and statistically significant reduction in BMI, decreasing from 31.3 ± 5.3 kg/m^2^ at admission to 28.2 ± 4.3 kg/m^2^ 6 months post‐discharge, corresponding to a total reduction of 3.1 kg/m^2^ (*p* < 0.001) [70]. This sustained decline highlights liraglutide's utility in weight loss and achieving long‐term BMI improvements among individuals with elevated baseline adiposity [70].

Reduction in waist circumference is also reported in several clinical trials since it is an important outcome associated with liraglutide treatment. In the LEAD trials, notably LEAD‐2 and LEAD‐3, participants demonstrated significant decreases in waist circumference alongside weight loss. For instance, liraglutide treatment was associated with statistically significant reductions in waist circumference compared to glimepiride treatments, indicating its role in reducing abdominal adiposity, which is a key risk factor for metabolic syndrome [67]. Specifically, reductions in waist circumference have been linked to improvements in insulin sensitivity and overall metabolic health [71].

#### 4.2.3 Tirzepatide

Tirzepatide has been studied in the randomized SURPASS clinical trial program, where participants experienced weight reductions ranging from 5.3 kg to 12.4 kg and from 6.5 kg to 17.1 kg, depending on dose and comparator (*p* < 0.05). For example, SURPASS‐5 reported mean body‐weight changes of −5.4 kg (5 mg), −7.5 kg (10 mg) and −8.8 kg (15 mg) versus placebo, and SURPASS‐2 and other SURPASS trials reported larger absolute reductions versus active comparators [72]. These reductions were observed across 5 mg, 10 mg and 15 mg doses, with greater effects at higher doses and in comparison to agents such as semaglutide 1 mg, insulin degludec and insulin glargine [73]. Weight loss was generally consistent regardless of gastrointestinal symptoms, with reductions ranging from −6.2 to −14.9 kg in those reporting symptoms and −6.2 to −13.3 kg in those who did not (*p* < 0.01) [74].

A post hoc analysis of the SURPASS trials identified a modest but statistically significant correlation between weight loss and HbA1c reduction (correlation coefficients: 0.1438–0.3130) [75]. Additionally, an incremental response in time spent with ≥ 5% body weight reduction were observed (20%–77% with tirzepatide vs. 25% with semaglutide 1 mg; *p* < 0.001). Consistent with these findings, Rosenstock et al. [72] in the SURPASS‐1 trial reported that tirzepatide induced dose‐dependent reductions in body weight ranging from −7.0 kg to −9.5 kg after 40 weeks of treatment in individuals with type 2 diabetes. Similarly, Aronne et al. [76] demonstrated in the randomized SURMOUNT‐4 trial that participants with obesity maintained mean weight reductions of approximately 20.9% at 88 weeks during continued tirzepatide therapy, reflecting its sustained efficacy in weight management [76]. Comparable trends have been observed with GLP‐1 receptor agonists, including liraglutide and semaglutide, though correlation coefficients varied (e.g., 0.248 for liraglutide, −0.04 to 0.32 for semaglutide) [77, 78].

While clinical trial data consistently highlight tirzepatide's profound impact on mass reduction, real‐world evidence underscores the synergistic role of lifestyle quality on pharmacological outcomes. Emerging data indicate that concurrent adherence to a structured Mediterranean diet significantly enhances tirzepatide's therapeutic footprint over a 3‐month period, driving superior reductions in visceral adiposity indices and marked improvements in fasting insulin and HOMA‐IR independent of age, gender or baseline BMI [79].

BMI reductions were also observed in a phase 3 SURPASS post hoc analysis comparing tirzepatide (5, 10 and 15 mg) to dulaglutide (0.75 mg) in Japanese patients with type 2 diabetes. The impact of tirzepatide was stratified across BMI subgroups. In individuals with BMI < 25 kg/m^2^, the proportion meeting the composite metabolic endpoint declined markedly by week 52, from 43.2% to 13.3% with tirzepatide 5 mg, 42.9% to 12.9% with 10 mg and 42.9% to 0.0% with 15 mg [80]. By contrast, participants with BMI 25 to < 30 kg/m^2^ and ≥ 30 kg/m^2^ showed higher baseline prevalence of metabolic abnormalities (≥ 71.2% and ≥ 81.4%, respectively), with notable improvements following tirzepatide administration. In the ≥ 30 kg/m^2^ group, endpoint attainment decreased from 86.7% to 57.1% with 5 mg, 81.4% to 42.5% with 10 mg and 81.4% to 28.2% with 15 mg, indicating increased dose–response improvements in metabolic parameters in individuals with elevated BMI [80].

In a post hoc analysis of multiple phase 3 trials encompassing East Asian participants (SURPASS‐1, −3, −4, −5, J‐mono and J‐combo) [81], tirzepatide administration resulted in incremental improvements at week 52 in key cardiometabolic parameters, including BMI, and waist circumference across both BMI (< 25 and ≥ 25 kg/m^2^) stratifications. These changes were accompanied by favourable shifts in insulin sensitivity, lipid metabolism and blood pressure regulation.

Additionally, a sub study involving 296 patients with type 2 diabetes from the SURPASS‐3 trial revealed that tirzepatide (10 mg and 15 mg pooled treatment group) significantly reduced hepatic fat and lowered volumes of both visceral and subcutaneous abdominal adipose tissue compared with insulin degludec [82]. Reductions in waist circumference were notably proportional to dose in the tirzepatide treatment arms, with mean decreases of −14.0 cm, −17.7 cm and −18.5 cm observed with the 5 mg, 10 mg and 15 mg doses, respectively, compared to −4.0 cm in the placebo group. The estimated differences relative to placebo were −10.1 cm for 5 mg, −13.8 cm for 10 mg and −14.5 cm for 15 mg, indicating its efficacy across escalating dose levels [82, 83].

Furthermore, the comparative clinical landscape between these agents extends into cardiovascular hemodynamics. While the SURPASS‐2 trial established tirzepatide's superiority over semaglutide regarding HbA1c and weight reduction, secondary insights from the broader SURPASS clinical program (including SURPASS‐1 and SURPASS‐3) reveal a robust reduction in mean systolic blood pressure, lowering readings by up to 6.6 mmHg. This clinical benefit likely interfaces with underlying cellular mechanisms unique to dual‐incretin pathways: specifically, preclinical data demonstrate that GIP receptor signalling actively mitigates peripheral arterial remodelling and fosters endothelial regeneration via nitric oxide synthase activation. Consequently, the dual‐agonist paradigm may offer a multifaceted therapeutic edge in reducing both metabolic and vascular burden over traditional monotherapies [84].

**FIGURE 3:**
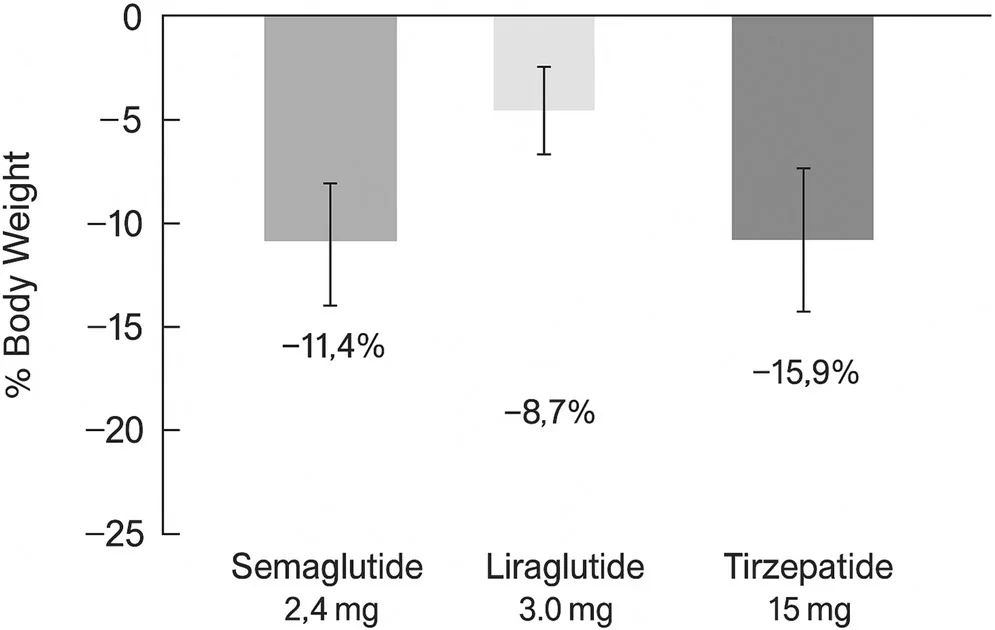
Comparative weight‐loss effects of semaglutide, liraglutide and tirzepatide. Bar graph illustrating percentage body‐weight reduction achieved with semaglutide 2.4 mg, liraglutide 3.0 mg and tirzepatide 15 mg at therapeutic doses for obesity management. Semaglutide is associated with mean reductions of approximately 10%–15%, liraglutide with 5%–7%, and tirzepatide with the greatest reductions, ranging from 15%–21% as demonstrated across pivotal STEP, SCALE and SURMOUNT clinical programs. Detailed data sources and methodological notes for this figure are provided in File S1, Figure S2.

Semaglutide, liraglutide and tirzepatide have all demonstrated significant weight reduction in individuals with type 2 diabetes, with tirzepatide generally producing the greatest absolute reductions across separate clinical trials (Table 3, Figure 3). Variations in reported outcomes may be attributed to differences in baseline BMI, treatment duration, dosing regimens and study populations, as well as factors such as concomitant therapies, lifestyle interventions, adherence and baseline metabolic status. Differences in trial design, including endpoint definitions, comparator agents and follow‐up duration, may further influence the magnitude of observed weight change.

**TABLE 3:** Comparative effects of semaglutide, liraglutide and tirzepatide on weight, BMI and waist circumference across major clinical trials.

Agent	Trials	Dose (mg)	Weight reduction	BMI reduction	Waist circumference reduction	Duration (weeks)
Semaglutide	SUSTAIN, STEP, SELECT	0.5–2.4 (SC, weekly) 3–14 (oral, daily)	7.9%–14.9%	1.5–4.5 kg/m^2^	7.7–9.7 cm	30–208
Liraglutide	LEAD, meta‐analyses	0.3–3.0 (daily)	1.8–4.91 kg 7.4%–10%	0.93–3.1 kg/m^2^	3.55–4.3 cm	3–26
Tirzepatide	SURPASS, Post Hoc SURMOUNT	5–15 (weekly)	5.3–17.1 kg 11.0%–20.9%	2–6 kg/m^2^	3.8–11.5 cm	28–88

Although weight reduction is associated with improvements in cardiometabolic risk, these findings reflect surrogate outcomes and do not directly establish cardiovascular benefit. As these results are derived from separate trials with heterogeneous designs and populations, comparisons across agents should be interpreted as indirect and hypothesis‐generating.

From a clinical perspective, these differences are particularly relevant in patients with obesity or weight‐driven cardiometabolic risk, where greater weight reduction may contribute to improvements in insulin resistance and metabolic health and may influence treatment selection when weight loss is a primary therapeutic goal.

### 4.3 Lipid Profile Modulation

**FIGURE 4:**
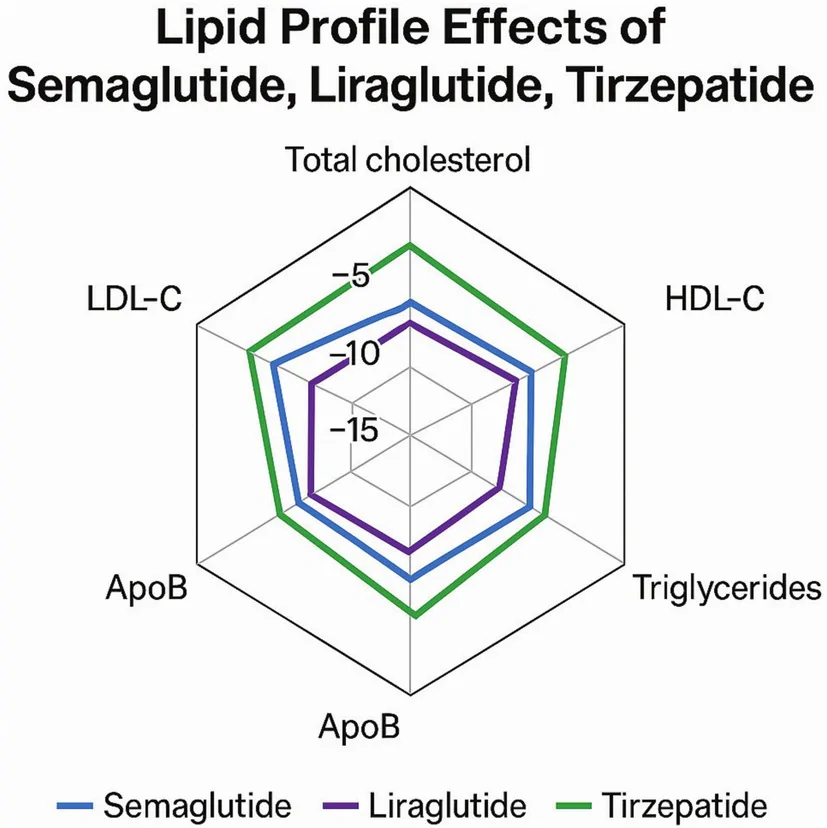
Normalized lipid profile effects of semaglutide, liraglutide and tirzepatide. This radar chart illustrates the relative directional improvements in lipid parameters, including total cholesterol, LDL cholesterol, HDL cholesterol, triglycerides and apolipoprotein‐based on aggregate findings from randomized clinical trials and meta‐analyses. Data were normalized to qualitative effect magnitudes (slight, mild, moderate, substantial) derived from Table 4 of the manuscript. Tirzepatide demonstrates the strongest overall lipid modulation, particularly in triglyceride and ApoB reduction, whereas semaglutide and liraglutide show modest but consistent improvements across most parameters. Detailed data sources and methodological notes for this figure are provided in File S1, Figure S3.

Incretin‐based therapies have been investigated for their effects on lipid metabolism in individuals with obesity and type 2 diabetes [65]. Evidence from clinical trials and meta‐analyses suggests that semaglutide, liraglutide and tirzepatide may influence lipid parameters, including total cholesterol, triglycerides (TG) and low‐density lipoprotein cholesterol (LDL‐C) [65, 85], though the magnitude and consistency of these effects vary across agents and populations. These effects are summarized visually in Figure 4.

#### 4.3.1 Semaglutide

Findings from the STEP clinical trials provide validation of semaglutide's efficacy in altering lipid metabolism. In a systematic review including a large cohort of participants, weekly administration of semaglutide was associated with significant changes in fasting lipid profiles. Compared to control groups, semaglutide decreased the percentage levels of total cholesterol, LDL, VLDL, free fatty acids and triglycerides, while leading to a modest increase in HDL. These lipid modifications underscore semaglutide's role in improving patient lipid profile in obese, non‐diabetic patients [65]. Similarly, a retrospective analysis indicated considerable improvements in lipids among diabetic patients, including declines in total cholesterol and non‐HDL‐C, suggesting a potential role for semaglutide as a potent therapeutic agent in managing dyslipidemia in non‐diabetic patients [86].

In a comprehensive assessment by Katsuyama et al. [87], semaglutide has been shown to stimulate insulin action, reduce the influx of free fatty acids (FFA) to the liver, and enhance lipoprotein lipase (LPL) activity. These mechanisms contributed to favourable changes in the lipoprotein profile, including reductions in intermediate‐density lipoprotein (IDL) and very low‐density lipoprotein (VLDL), which were reflected as significant decreases in non‐HDL cholesterol. Consistent with earlier findings, semaglutide was also associated with improvements in LDL‐C, HDL‐C and triglyceride levels in diabetic, obese patients with high prevalence of metabolic dysfunction associated steatotic liver disease (MASLD) [87]. Collectively, these lipid profile changes have been associated with cardiovascular outcomes in prior studies, as previously demonstrated in the SUSTAIN 6 trial [87]. This mechanism suggests a multifaceted approach where semaglutide can lower lipid levels and target the underlying metabolic dysfunction prevalent in patients with obesity and type 2 diabetes.

In addition, the data presented by Li and Liu highlight that semaglutide positively affects biomarkers associated with MASLD, illustrating a beneficial role in lipid regulation by ameliorating liver function markers, including alanine aminotransferase (ALT) and aspartate aminotransferase (AST) [88]. This improvement in liver health is crucial, as dysregulated lipid profiles in MASLD can exacerbate metabolic complications [88].

Notably, oral semaglutide has also been observed to significantly influence lipid metabolism in various populations, showcasing reductions in apolipoprotein B levels and postprandial TG levels following a high‐fat meal in a randomized, double‐blind study with obese, non‐diabetic patients and an observational study including diabetic and obese individuals [86, 89]. Suggesting that semaglutide can potentially alter both fasting lipid levels and impact lipid absorption and metabolism after dietary intake, which is pivotal for comprehensive metabolic control.

#### 4.3.2 Liraglutide

Moving to liraglutide, evidence suggests that it is associated with favourable changes in lipid profiles. A systematic review and meta‐analysis indicated that liraglutide results in important reductions in triglycerides, total cholesterol and LDL‐C, particularly at a 1.2 mg daily dosage in patients who were both diabetic and obese [90]. The higher 1.8 mg dosage was noted to have less significant lipid profile improvements, with no major difference observed compared to placebo, indicating dose‐dependent variations in efficacy that warrant further exploration [90].

A cohort study involving overweight and obese individuals with T2DM undergoing at least 3 months of stable metformin therapy assessed the impact of adjunctive liraglutide treatment at doses up to 3 mg/day. The analysis revealed a statistically significant reduction in the total cholesterol to HDL ratio (−0.20 log units; *p* = 0.020). Additionally, non‐significant but suggestive decreases were observed in total cholesterol/high‐density lipoprotein cholesterol ratio, indicating potential improvements in the lipid profile associated with liraglutide administration [91].

In another study, a short‐term randomized controlled trial involving sub‐Saharan patients with uncontrolled type 2 diabetes, liraglutide add‐on therapy (1.2 mg/day for 2 weeks) led to a significant reduction in LDL cholesterol levels, decreasing from 0.85 g/L to 0.54 g/L (*p* = 0.04). This lipid‐lowering effect was not observed with vildagliptin, indicating a potential lipid‐lowering effect on the lipid profile within a brief intervention period. These findings contribute to the growing evidence supporting the cardiometabolic advantages of GLP‐1 receptor agonists in high‐risk populations [92].

Another clinical study specifically assessed liraglutide's effects on overweight and obese patients without overt diabetes, revealing reductions in remnant cholesterol and apolipoproteins, particularly apoB [93]. This aligns with findings from Engelbrechtsen et al. [94], who reported that long‐term liraglutide treatment lowered  apoB levels and improved the atherogenic lipid profile, thereby contributing to decreased cardiovascular mortality among treated individuals.

Moreover, liraglutide has demonstrated efficacy in ameliorating postprandial lipid levels, specifically after high‐fat meals. A randomized controlled trial including individuals with well‐controlled T2DM highlighted that liraglutide suppressed postprandial triglyceride and apolipoprotein B48 elevations, supporting its beneficial effects on lipid metabolism relative to meal intake [95]. This could be particularly important for patients at risk of postprandial dyslipidemia.

#### 4.3.3 Tirzepatide

Tirzepatide, as a dual agonist, has also been shown to effectively modulate lipid profiles in patients with T2DM through several mechanisms. In a randomized controlled trial Wilson et al. [96] reported dose‐dependant reductions in triglycerides and apoB levels following tirzepatide treatment. Specifically, the study demonstrated that diabetic and obese patients receiving tirzepatide experienced a significant decrease in large triglyceride‐rich lipoproteins and small LDL particles. These findings support the cardiovascular risk modulation of tirzepatide beyond glucose regulation by improving lipid biomarkers associated with insulin resistance and cardiovascular risks. Furthermore, in a post hoc analysis of a Phase 2 RCT, Zhou et al. [97] reported that tirzepatide significantly reduced appetite and food intake, which contributes to its overall effect on improving lipid profiles and body weight in diabetic patients. In this review, data from two randomized controlled trials comparing tirzepatide with placebo or active comparators demonstrated significant improvement in lipid metabolism across all dosing groups over 26–40 weeks [97].

Meta‐analytic estimates confirmed dose‐dependent reduction in triglyceride levels across all tirzepatide doses. In contrast, the effects on LDL cholesterol were inconsistent and generally not superior to comparator therapies, except for modest reductions reported in the 5 mg subgroup. Importantly, HDL cholesterol levels increased substantially following tirzepatide treatment, further underscoring its favourable influence on the atherogenic lipid profile. However, due to limited data availability, outcomes related to other types of lipids such as total cholesterol could not be robustly assessed [97].

An interesting aspect of tirzepatide's action is highlighted by the systematic review and meta‐analysis from Cai et al. [98], which indicated its efficacy in metabolically unhealthy obese patients. These patients exhibited improvements in insulin sensitivity, reduced fatty acid synthesis and regulation of lipid metabolism, resulting in lowered blood pressure and enhanced cardiovascular health. Such mechanisms suggest that tirzepatide may be particularly effective in addressing the lipid dysregulation prevalent in this demographic.

Furthermore, findings from the systematic review conducted by Mahar et al. [99] emphasized the significance of tirzepatide in correcting dyslipidemia in patients with obesity, diabetes and metabolic syndrome. The review concluded that improvements in lipid metabolism would lead to a reduction in the risk of atherosclerotic cardiovascular diseases, suggesting a possible contribution of tirzepatide's role in comprehensive diabetes management.

Meta‐analytic data revealed improvements across key lipid parameters that are proportional to dose, including significant reductions in total cholesterol (−4.77% to −6.55%), LDL‐C (−5.60% to −7.82%) and triglycerides (−13.18% to −22.08%), while HDL‐C levels increased markedly with escalating doses (3.78%–6.94%) [99]. These findings were consistent across multiple cohorts and support tirzepatide's multifaceted benefit in modulating atherogenic lipoproteins, particularly at higher doses, compared to placebo, insulin and GLP‐1 receptor agonists [99]. Additionally, a post hoc analysis by Onishi et al. [80] reported that tirzepatide reduced the prevalence of metabolic abnormalities, including serum lipids, in Japanese patients with T2D. These findings provide supportive evidence in a specific population; however, generalizability across broader and more diverse populations remains to be established.

Semaglutide, liraglutide and tirzepatide have each demonstrated varying degrees of impact on lipid profiles in individuals with type 2 diabetes and obesity (Table 4). Semaglutide is associated with modest reductions in total cholesterol, LDL‐C and triglycerides, alongside slight increases in HDL‐C, with effects observed in both fasting and postprandial states. Liraglutide shows mild to moderate improvements, particularly in triglycerides and apoB levels, with evidence of dose‐dependent variability. Tirzepatide appears to exert more pronounced lipid‐lowering effects, especially in reducing triglycerides and apoB, with consistent HDL‐C elevation and moderate reductions in total and LDL cholesterol across dosing groups.

**TABLE 4:** Comparative effects of semaglutide, liraglutide and tirzepatide on lipid parameters and mechanistic pathways.

Agent	Total cholesterol	LDL‐C	HDL‐C	Triglycerides	Mechanistic notes
Semaglutide	**↓ modest** (clinical data show small but consistent decreases (≈3%–6%))	**↓ modest** (reductions of ~5%–10%)	**↑ slight** (HDL rises are generally minimal (≈1%–3%))	**↓ moderate** (~10%–15%)	Reduces FFA influx, enhances LPL activity, improves postprandial lipid metabolism
Liraglutide	**↓ mild** ((≈2%–4%))	**↓ mild** (~5%–7%)	**↑ slight** (small, non‐significant HDL improvement)	**↓ moderate** (~10%–20%, especially at 1.2–1.8 mg)	Modulates lipolysis, enhances thermogenesis and lipid oxidation, reduces apoB and postprandial TGs
Tirzepatide	**↓ moderate** (Mean −4 to −7%)	**↓ variable** (Changes inconsistent across trials)	**↑ moderate** (Increases ~4%–7%, a moderate improvement vs. GLP‐1 RAs)	**↓ substantial** (reductions −13% to −22%)	Reduces large TG‐rich lipoproteins, improves insulin sensitivity, lowers apoB

*Note:* Magnitude descriptors (slight, mild, modest, moderate, substantial) correspond to approximate percentage changes observed across studies: slight < 5%, mild 5%–10%, moderate 10%–20%, substantial > 20%.

These effects have been confirmed in multiple randomized trials and meta‐analyses, with tirzepatide showing greater improvements in certain lipid parameters compared to placebo, insulin and GLP‐1 receptor agonists. The observed lipid modulation likely arises from distinct yet complementary mechanisms. However, the magnitude and consistency of these effects vary according to dose, treatment duration, baseline lipid status and patient characteristics. While all three agents contribute to favourable lipid modulation, these findings represent surrogate outcomes and their direct impact on cardiovascular events remains to be established.

From a clinical perspective, these lipid‐modifying effects may contribute to overall cardiovascular risk reduction; however, given the absence of consistent outcome data and the reliance on indirect comparisons across heterogeneous studies, these findings should be interpreted with caution.

## 5 Major Adverse Cardiovascular Events

Unlike glycaemic, weight and lipid outcomes, which are considered surrogate markers, MACE represents a confirmed clinical endpoint derived from dedicated cardiovascular outcome trials. Major adverse cardiovascular events (3‐point MACE), typically defined as a composite of cardiovascular death, nonfatal myocardial infarction (MI) and nonfatal stroke, serve as the primary endpoint in cardiovascular outcome trials (CVOTs) evaluating glucagon‐like peptide‐1 receptor agonists (GLP‐1 RAs).

### 5.1 Semaglutide

In the SUSTAIN‐6 trial, semaglutide demonstrated a 26% relative risk reduction in 3‐point MACE compared to placebo (HR 0.74; 95% CI 0.58–0.95), primarily driven by reductions in nonfatal stroke and nonfatal MI, while cardiovascular mortality remained statistically neutral. Notably, post hoc pooled analyses from SUSTAIN trials suggest a ~35% reduction in stroke incidence with semaglutide, positioning it as one of the strongest GLP‐1 RAs for cerebrovascular protection [100].

### 5.2 Liraglutide

In contrast, liraglutide demonstrated a 13% reduction in 3‐point MACE in the landmark LEADER trial (HR 0.87; 95% CI 0.78–0.97), with benefit largely attributed to significant reductions in cardiovascular death and nonfatal MI, while stroke reduction was not statistically significant [101]. This makes liraglutide particularly relevant for patients with high baseline cardiovascular mortality risk, especially those prioritizing survival endpoints. However, in real‐world combination settings, adding a GLP‐1 RA to a Sodium‐Glucose Cotransporter 2 (SGLT2) inhibitor in patients with concurrent Atherosclerotic Cardiovascular Disease (ASCVD) and heart failure significantly reduces 1‐year mortality and hospitalization, though subgroup analyses suggest this cardiorenal benefit is driven predominantly by agents like semaglutide, whereas liraglutide demonstrates a neutral effect in this dual‐therapy context [102].

### 5.3 Tirzepatide

While tirzepatide has not yet been evaluated in a dedicated CVOT at the time of writing, a pre‐specified meta‐analysis of MACE across pooled SURPASS trials (including SURPASS‐4, which enrolled a high‐risk population) reported a 27% relative risk reduction in 3‐point MACE (HR 0.73; 95% CI 0.51–1.05) and a 46% reduction in 4‐point MACE (including hospitalization for unstable angina), suggesting a potentially robust cardiovascular benefit. Confirmation is pending from the ongoing SURPASS‐CVOT, which will assess hard outcomes versus dulaglutide in patients with established cardiovascular disease [103]. Importantly, a narrative review in non‐diabetic obese individuals reports consistent improvements in multiple cardiometabolic risk markers including waist circumference, blood pressure and ASCVD risk with tirzepatide induced weight loss, supporting potential cardiovascular benefit beyond glycaemic control [103]. This clinical cardioprotection is supported by new in vitro evidence showing that tirzepatide directly modulates cardiomyocyte β‐adrenoceptor signalling and glucose metabolism via non‐canonical receptor cross‐talk, mitigating hyperglycemia and senescence‐induced cardiac dysfunction independent of classical incretin receptor action [104].

In addition to these peripheral cardiac and systemic vascular benefits, emerging basic science models indicate that tirzepatide's tissue protection extends directly into the cerebrovascular architecture. In preclinical models of ischemic stroke and oxygen–glucose deprivation, tirzepatide significantly ameliorates blood–brain barrier (BBB) dysfunction and reduces endothelial permeability. Mechanistically, this neuroprotective effect is driven by the activation of C/EBP‐α signalling, which restores the expression of critical tight junction proteins—specifically Claudin‐1—thereby preserving BBB structural integrity and mitigating acute post‐stroke neuronal damage [105].

When comparing individual MACE components, semaglutide offers the strongest evidence for stroke prevention, liraglutide for mortality‐driven benefit, and tirzepatide shows broad signals across all three endpoints, pending CVOT confirmation (Table 5) [100, 101, 103].

**TABLE 5:** Comparative cardiovascular outcomes of semaglutide, liraglutide and tirzepatide based on evidence from major clinical trials.

Parameter	Semaglutide	Tirzepatide	Liraglutide
Key trials/evidence	SUSTAIN 6 pooled post hoc analyses	Pooled SURPASS trials (meta‐analysis). SURPASS‐CVOT ongoing	LEADER
3‐point MACE outcome	26% reduced risk (HR 0.74; 95% CI 0.58–0.95)	27% reduced risk (HR 0.73; 95% CI 0.51–1.05)—not statistically significant yet	13% reduced risk (HR 0.87; 95% CI 0.78–0.97)
Primary drivers	Reduction in nonfatal stroke and MI. CV death neutral	Reduction across all MACE components; strongest in 4‐point MACE	Reduced CV death and MI. Stroke reduction not significant
Stroke impact	~35% reduction in stroke incidence (pooled SUSTAIN data)	Suggestive reduction; confirmation pending	No significant reduction
Cardiovascular death	Neutral	Signal present; not statistically confirmed	Statistically significant ↓
Clinical implication	Best evidence for stroke protection; ideal for cerebrovascular risk	Broad MACE reduction potential; confirmation pending; promising for high CV risk + CKD	Strongest mortality benefit; suitable for high CV mortality risk patients

## 6 Renal Function Markers

**FIGURE 5:**
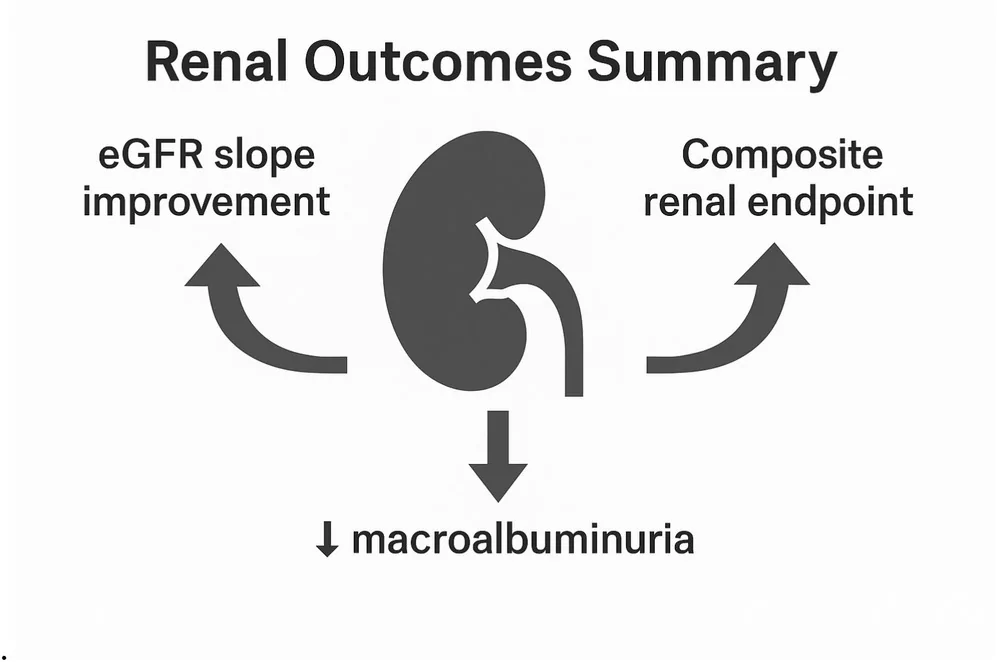
Renal outcomes summary. This figure illustrates the principal renal benefits associated with incretin‐based therapies, including attenuation of estimated glomerular filtration rate (eGFR) decline, reduced progression to macroalbuminuria and favourable effects on composite renal endpoints. These outcomes are depicted schematically using a central kidney graphic with directional indicators representing the relative improvements.

Changes in eGFR and progression of albuminuria are critical surrogate endpoints for renal disease in patients with T2DM and obesity. Both parameters are independently associated with cardiovascular events and all‐cause mortality, making them integral to assessing long‐term outcomes beyond glycaemic control. Figure 5 provides a visual summary of the primary renal endpoints, specifically the eGFR slope, progression of macroalbuminuria and composite renal outcomes.

### 6.1 Semaglutide

Semaglutide has consistently demonstrated renal benefits across multiple clinical programs. In the SUSTAIN‐6 trial, semaglutide significantly reduced the risk of new or worsening nephropathy by 36% (HR 0.64; 95% CI 0.46–0.88), largely driven by a reduction in new‐onset macroalbuminuria. In a post hoc analysis of SUSTAIN and PIONEER, semaglutide attenuated eGFR decline and reduced albuminuria progression, with the greatest benefits seen in participants with baseline micro‐ or macroalbuminuria with effects observed across HbA1c subgroups, suggesting some degree of glucose‐independent benefit [106]. Furthermore, in a pre‐specified secondary analysis of the SELECT trial, semaglutide slowed eGFR decline in non‐diabetic individuals with cardiovascular disease and obesity, reinforcing renal protection beyond glycaemic mechanisms [106].

### 6.2 Liraglutide

Liraglutide, in the LEADER trial, demonstrated a 22% relative risk reduction in new or worsening nephropathy (hazard ratio 0.78; 95% confidence interval 0.67–0.92), primarily driven by fewer cases of new‐onset persistent macroalbuminuria. Changes in eGFR over time did not differ significantly between groups, and no formal mediation analysis was reported. While mechanistic data suggest potential glucose‐independent pathways, the observed renal benefit appears largely attributable to improvements in glycaemic control, blood pressure and overall haemodynamic status rather than direct nephroprotective effects [8].

### 6.3 Tirzepatide

Tirzepatide has shown similarly promising renal signals in SURPASS‐4, which enrolled a high‐risk cardiovascular cohort with prevalent Chronic Kidney Disease (CKD). Across all doses, tirzepatide significantly attenuated the decline in eGFR and reduced new‐onset macroalbuminuria. A prespecified composite renal endpoint (≥ 40% eGFR decline, renal death, or macroalbuminuria) was favourably impacted. A causal mediation analysis indicated that only 40%–60% of the renal benefit was mediated through traditional factors (HbA1c, blood pressure, weight), suggesting additional glucose‐independent mechanisms [103].

Comparatively, tirzepatide provides the most consistent evidence for partially glucose‐independent renal protection, supported by robust mediation data (Table 6). Semaglutide's renal benefits in non‐diabetic populations also suggest an independent component, though not formally quantified. In contrast, liraglutide's renoprotection seems largely mediated by glycaemic improvement. These distinctions are clinically relevant when tailoring therapy for patients with CKD or high renal risk, irrespective of glycaemic targets.

**TABLE 6:** Comparative renal outcomes and mechanistic insights of semaglutide, liraglutide and tirzepatide in type 2 diabetes and chronic kidney disease.

Parameter	Semaglutide	Tirzepatide	Liraglutide
Key trials	SUSTAIN 6, PIONEER (pooled), SELECT	SURPASS 4	LEADER
eGFR trajectory	Slowed decline (SUSTAIN, SELECT); effect seen even in non‐diabetics	Slowed decline across all doses in CKD patients	No significant change
Albuminuria effects	↓ New‐onset macroalbuminuria (SUSTAIN 6); benefit in baseline micro‐/macroalbuminuria	↓ New‐onset macroalbuminuria; strong impact on composite renal outcome	↓ Persistent macroalbuminuria (LEADER)
Composite renal outcome	36% ↓ risk of new/worsening nephropathy (HR 0.64; 95% CI 0.46–0.88)	↓ Risk of ≥ 40% eGFR decline, renal death or macroalbuminuria	22% ↓ risk of new/worsening nephropathy (HR 0.78; 95% CI 0.67–0.92)
Glucose‐independent effects	Suggested (SELECT, PIONEER subgroup analysis), not formally quantified	Supported by mediation analysis (40%–60% not explained by HbA1c, BP or weight)	Likely minimal; effect appears mediated by glycaemic and haemodynamic changes
Non‐diabetic renal data	Yes—SELECT trial supports benefit in obese, non‐diabetic patients	No dedicated non‐diabetic renal trial, though CKD cohort included	No data in non‐diabetic populations
Clinical implication	Effective in both diabetic and non‐diabetic CKD; possible glucose‐independent protection	Strongest mechanistic evidence for non‐glycaemic renal benefit; ideal in diabetic CKD	Appropriate for renal risk reduction primarily through glycaemic control

## 7 Safety and Tolerability

All three incretin‐based therapies—semaglutide, liraglutide and tirzepatide—are generally well tolerated, with gastrointestinal (GI) symptoms representing the most commonly reported adverse effects across agents. Tirzepatide has been associated with a higher frequency of gastrointestinal adverse events compared with GLP‐1 receptor agonists, particularly at higher doses. Nausea, vomiting, diarrhoea and constipation typically occur during dose escalation with tirzepatide producing the highest frequency of GI events, followed by semaglutide and then liraglutide. Gradual titration remains essential to improving tolerability and reducing early discontinuation rates.

Risks of gallbladder‐related events, including cholelithiasis and cholecystitis, have been observed with all GLP‐1/GIP‐based therapies, largely associated with rapid weight loss. Although the absolute risk is low, clinicians should remain vigilant in patients with a prior history of gallstones. The risk of pancreatitis is rare but has been reported; current evidence does not demonstrate a causal relationship, yet these agents should be used cautiously in individuals with a prior pancreatitis history.

Hypoglycemia is uncommon with GLP‐1 or GIP/GLP‐1 therapy alone and primarily occurs when treatment is combined with insulin or sulfonylureas. Additional adverse events include injection‐site reactions, mild increases in heart rate, and transient gastrointestinal discomfort. All agents are contraindicated in individuals with a personal or family history of medullary thyroid carcinoma or multiple endocrine neoplasia type 2 (MEN2).

Overall, semaglutide, liraglutide and tirzepatide maintain favourable safety profiles and are well tolerated in diverse patient populations, with manageable side effects that can be minimized through individualized dose titration and careful patient selection.

## 8 Conclusion

Semaglutide, liraglutide and tirzepatide each provide clinically meaningful improvements in cardiometabolic risk markers, including glycaemic control, body weight reduction and lipid parameters, with differences in magnitude, consistency and dose–response relationships across agents. Tirzepatide demonstrates the greatest reductions in HbA1c and body weight across phase 3 trials, while semaglutide shows substantial and consistent effects across both diabetes and obesity populations. Liraglutide, although comparatively less potent, remains effective and is supported by a well‐established safety and tolerability profile. Variability in the outcomes reflects differences in baseline characteristics, dosing regimens and populations across trials, with no direct head‐to‐head comparisons available.

With respect to cardiovascular outcomes, semaglutide and liraglutide demonstrated reductions in major adverse cardiovascular events in large CVOT, supporting their role in patients with established cardiovascular disease or high cardiovascular risk. Data for tirzepatide remain limited to secondary analyses, and definitive conclusions are awaited from dedicated CVOTs.

Similarly, renal outcomes differ in both magnitude and mechanistic interpretation. Semaglutide and liraglutide reduce composite renal endpoints, primarily driven by decreases in albuminuria, with semaglutide additionally attenuating eGFR decline, including in non‐diabetic populations, suggesting partial glucose‐independent effects. In contrast, liraglutide's benefits appear primarily mediated by glycaemic and haemodynamic improvements. Tirzepatide demonstrated slowed eGFR decline and reduced macroalbuminuria, with mediation analyses indicating a substantial glucose‐independent component. Tirzepatide may provide the most consistent evidence for partially glucose‐independent renal protection, although confirmation from dedicated renal outcome trials is required.

Overall, while all three agents improve cardiometabolic profiles, these findings are based on indirect cross‐trial comparisons rather than head‐to‐head randomized studies; therefore, differences in efficacy and outcomes should be interpreted cautiously and not as definitive evidence of comparative superiority. Given that the available evidence is derived from heterogeneous clinical trials with differing designs, populations and endpoints, therapeutic decisions should be individualized, according to comorbidities, treatment goals, tolerability, dosing preferences, renal function and prior therapy exposure.

Despite the accumulating evidence, important gaps remain. Direct head‐to‐head trials, particularly comparing tirzepatide with higher dose semaglutide are needed to better define their relative efficacy and safety profiles. Long‐term cardiovascular and renal outcome data for tirzepatide are still pending, and evidence in underrepresented populations remains limited. Additionally, further advances in drug formulations, particularly improved oral incretin therapies, may enhance accessibility and adherence.

Addressing these gaps will be essential to refine clinical decision‐making and optimize therapeutic strategies in the management of type 2 diabetes, obesity and cardiometabolic disease.

## 9 Limitations

A key limitation of this review is the reliance on indirect cross‐trial comparisons in the absence of head‐to‐head randomized studies. Differences in study design, baseline characteristics, outcome definitions and follow‐up durations limit the ability to draw definitive conclusions regarding comparative efficacy and safety between agents.

Second, variability in baseline factors, including HbA1c, body mass index, cardiovascular risk, renal function, background therapies, dosing regimens and formulations limits standardization across outcomes.

Third, lipid parameters and mechanistic biomarkers were not consistently defined or measured across studies, with variability in lipid measurement methods, assay techniques and outcome definitions across trials, which may limit comparability of reported lipid outcomes.

Fourth, the level of evidence differs across domains. Cardiovascular outcomes for semaglutide and liraglutide are supported by dedicated CVOTs, whereas evidence for tirzepatide is derived from secondary analyses pending ongoing trials. Renal outcomes are similarly based on secondary analyses.

Finally, inclusion of randomized trials, meta‐analyses and observational studies introduces variability in evidence quality and potential bias, as no formal weighting or hierarchy of evidence was applied. The availability of long‐term outcomes, real‐world effectiveness data and evidence in underrepresented populations remains limited.

## Author Contributions

**Hiba Bawadi:** conceptualization, validation, supervision, project administration, writing – review and editing. **Haya Abuhijleh:** writing – original draft, writing – review and editing, methodology, validation. **Mamon Nofal:** writing – original draft, writing – review and editing, validation. **Zain Z. Zakaria:** conceptualization, validation, supervision, writing – review and editing. **Maha Al‐Asmakh:** conceptualization, supervision, visualization, validation, writing – review and editing, funding acquisition.

## Conflicts of Interest

The authors declare no conflicts of interest.

## Supporting information


**File S1:** edm270338‐sup‐0001‐FileS1.docx.

## Data Availability

The data that supports the findings of this study are available in the Supporting Information of this article.
