# Supplementary material for: Impact of Semaglutide, Liraglutide and Tirzepatide on Cardiometabolic Outcomes: A Comparative Narrative Review
Source: Endocrinol Diabetes Metab. 2026 Sep 16;9(5):e70338. doi: 10.1002/edm2.70338 (PMC13580159; doi:10.1002/edm2.70338)
Supplement: Supplementary file 1 [file EDM2-9-e70338-s001.docx]

**Supplementary file 1**

**Figure 1. Comparative HbA1c Reductions With Semaglutide, Liraglutide, and Tirzepatide.**

Forest plot illustrating the range of HbA1c reductions reported in pivotal clinical trials of semaglutide, liraglutide, and tirzepatide. Semaglutide achieved reductions of approximately −1.3% to −2.0%, liraglutide produced reductions of −0.7% to −1.5%, and tirzepatide demonstrated the largest reductions, ranging from −2.0% to −2.3%. Estimates are derived from the SUSTAIN and PIONEER (semaglutide), LEAD (liraglutide), and SURPASS (tirzepatide) clinical development programs.

The ranges were taken **directly from the reductions reported in the major clinical trial programs** for each drug:

**1- Semaglutide: –1.3% to –2.0%**

**Source:**

**SUSTAIN 1–5 trials** (injectable)

**PIONEER trials** (oral)

Your own manuscript text reports:

SUSTAIN 1: –1.45% to –1.55%

SUSTAIN 2: –1.30% and –1.60%

SUSTAIN 3–5: –1.21% to –1.80%

Oral semaglutide: up to –2.0% (PIONEER)

Therefore, the **overall clinically observed range** is:
**–1.3% to –2.0%**

**2-** **Liraglutide: –0.7% to –1.5%**

**Source:**

**LEAD 1–6 trials**
Your manuscript text states:

LEAD-2: –0.7% to –1.0%

LEAD-3: –0.84% to –1.14%

LEAD-4: –1.5%

LEAD-5: –1.33%

LEAD-6: –1.12%

Real-world: –0.9% to –2.2% (but typical clinical trial range is narrower)

The commonly accepted range in reviews and LEAD data is:
**–0.7% to –1.5%**

**3- Tirzepatide: –2.0% to –2.3%**

**Source:**

**SURPASS trials** (especially SURPASS-2)

Your text notes:

SURPASS-2: –2.01%, –2.24%, –2.30%

Across SURPASS: consistent –2.0% to –2.3%

The widely cited range is:
**–2.0% to –2.3%**

**Figure 2. Comparative Weight-Loss Effects of Semaglutide, Liraglutide, and Tirzepatide.**

Values extracted from table 3

**Figure 3. Normalized Lipid Profile Effects of Semaglutide, Liraglutide, and Tirzepatide.**
This radar chart illustrates the relative directional improvements in lipid parameters—including total cholesterol, LDL cholesterol, HDL cholesterol, triglycerides, and apolipoprotein-B—based on aggregate findings from randomized clinical trials and meta-analyses. Data were normalized to qualitative effect magnitudes (slight, mild, moderate, substantial) derived from Table 4 of the manuscript. Tirzepatide demonstrates the strongest overall lipid modulation, particularly in triglyceride and ApoB reduction, whereas semaglutide and liraglutide show modest but consistent improvements across most parameters.

**Normalization Method (based on Table 4)**

Converted descriptors → numerical scale:

Descriptor Assigned Value Slight (<5%)0.2Mild (5–10%)0.4Moderate (10–20%)0.7Substantial (>20%)1.0

Applied to Table 4 data

**Semaglutide**

Total Cholesterol → *modest* → **0.4**

LDL-C → *modest* → **0.4**

HDL-C → *slight increase* → **0.2**

TG → *moderate* → **0.7**

ApoB → (not explicitly listed → direction same as TC/LDL) → **0.4**

**Liraglutide**

Total Cholesterol → *mild* → **0.4**

LDL-C → *mild* → **0.4**

HDL-C → *slight increase* → **0.2**

TG → *moderate* → **0.7**

ApoB → *mild decrease* → **0.4**

**Tirzepatide**

Total Cholesterol → *moderate* → **0.7**

LDL-C → *variable mild–moderate* → **0.5**

HDL-C → *moderate increase* → **0.7**

TG → *substantial* → **1.0**

ApoB → *moderate decrease* → **0.7**

This is exactly what was plotted in Figure 4.
